# Nest Grouping Patterns of Bonobos *(Pan paniscus)* in Relation to Fruit Availability in a Forest-Savannah Mosaic

**DOI:** 10.1371/journal.pone.0093742

**Published:** 2014-04-02

**Authors:** Adeline Serckx, Marie-Claude Huynen, Jean-François Bastin, Alain Hambuckers, Roseline C. Beudels-Jamar, Marie Vimond, Emilien Raynaud, Hjalmar S. Kühl

**Affiliations:** 1 Behavioural Biology Unit, University of Liege, Liege, Belgium; 2 Conservation Biology Unit, Royal Belgian Institute of Natural Sciences, Brussels, Belgium; 3 Ecole Régionale Post-Universitaire d'Aménagement et de Gestion Intégrés des Forêts et Territoires Tropicaux, University of Kinshasa, Kinshasa, Democratic Republic of the Congo; 4 Department of Primatology, Max Planck Institute for Evolutionary Anthropology, Leipzig, Germany; 5 Landscape Ecology and Vegetal Production Systems Unit, Université Libre de Bruxelles, Brussels, Belgium; 6 Biodiversity and Landscape Architecture Unit, Gembloux AgroBio-Tech, University of Liege, Gembloux, Belgium; 7 German Centre for Integrative Biodiversity Research, Leipzig, Germany; Institut Pluridisciplinaire Hubert Curien, France

## Abstract

A topic of major interest in socio-ecology is the comparison of chimpanzees and bonobos' grouping patterns. Numerous studies have highlighted the impact of social and environmental factors on the different evolution in group cohesion seen in these sister species. We are still lacking, however, key information about bonobo social traits across their habitat range, in order to make accurate inter-species comparisons. In this study we investigated bonobo social cohesiveness at nesting sites depending on fruit availability in the forest-savannah mosaic of western Democratic Republic of Congo (DRC), a bonobo habitat which has received little attention from researchers and is characterized by high food resource variation within years. We collected data on two bonobo communities. Nest counts at nesting sites were used as a proxy for night grouping patterns and were analysed with regard to fruit availability. We also modelled bonobo population density at the site in order to investigate yearly variation. We found that one community density varied across the three years of surveys, suggesting that this bonobo community has significant variability in use of its home range. This finding highlights the importance of forest connectivity, a likely prerequisite for the ability of bonobos to adapt their ranging patterns to fruit availability changes. We found no influence of overall fruit availability on bonobo cohesiveness. Only fruit availability at the nesting sites showed a positive influence, indicating that bonobos favour food ‘hot spots’ as sleeping sites. Our findings have confirmed the results obtained from previous studies carried out in the dense tropical forests of DRC. Nevertheless, in order to clarify the impact of environmental variability on bonobo social cohesiveness, we will need to make direct observations of the apes in the forest-savannah mosaic as well as make comparisons across the entirety of the bonobos' range using systematic methodology.

## Introduction

Nest-building is an important behavioural feature shared by all species of great apes and is considered to be a basal adaptation underlying the aptitude of great apes for manipulating objects in their environment. The deep ancestry of this trait has possible implications for our understanding of the cognitive evolution of early hominoids [1], as it permits higher-quality sleep by providing thermoregulation [2], [3], reduced vulnerability to predators [2], [4], [5], more comfortable sleeping postures [4], [6], [7], and protection against pathogens [2], [4], [8]. The impact of environmental factors on the location of great ape nests has been the subject of a number of studies [6], [9]–[18], and nest counts are frequently used to estimate ape population density [19]–[29]. However the functionality of great ape nesting sites in relation to the dynamics of their social organization has been much less well-documented [1]. Bonobo nesting behaviour has not been as thoroughly investigated compared to that of chimpanzees [6], [16], [30], [31]. Nonetheless, several studies have already shown that nesting patterns could play an important role in their social behaviour. Fruth and Hohmann suggested that the aggregation of bonobos at nest sites at night could facilitate information transfer on the quality of food patches visited during the day [1], and that nests could serve as ‘taboo zones’ which can help bonobos avoid conflicts with group members [32]. Variation in the size and location of nest groups could reflect differences in social organisation and could provide us with insight into the species-specific elements of bonobo social structure [1].

Comparisons between the social organization of bonobos and chimpanzees have been made using data from a number of habituated populations and show that bonobos live in more cohesive communities and with a larger relative party size (i.e., the percentage of the total community size) [33]–[36]. The composition of chimpanzee parties changes more frequently than that of bonobos. Individual chimpanzees, usually adult females with infants, more often travel at a distance from the main parties, whereas bonobo parties usually range in the same general area and tend to aggregate towards the evening [37]. This trait is typical of all bonobo communities studied to date and thus appears to be characteristic of the species (for a review see Furuichi 2009 [37]), and numerous socio-ecological and environmental factors have been suggested to explain it: prolonged oestrus of bonobo females [38], close association between mothers and their adult sons [39], strong social bonds between females [40], high female social status [39], [41], food patch size [30], [42], availability of terrestrial herbaceous vegetation [43], and a feed-as-you-go foraging strategy (i.e., foraging during travel between fruit patches) [44]. A number of authors have interpreted the evidence to imply a difference in the nature of the fission-fusion social structure in the two species [37]. This might suggest that the grouping patterns of chimpanzees and bonobos have evolved through a process of long-term ecological and behavioural adaptations rather than merely reflecting a flexible response to current environmental differences. However, Boesch pointed out that chimpanzee grouping patterns in Taï (Ivory Coast) were similar to those of bonobos inhabiting similar rainforest study sites [34]. This finding supports the fact that we need social and ecological data for much of the bonobos' habitat, including the forest-savannah environment, which will render possible a socio-ecological comparison of both species across their ranges [37].

Until now, socio-ecological data on bonobos has been available only from dense tropical forests. While chimpanzees have been known for decades to live in savannahs, bonobo distribution was thought to be limited to dense rainforests. This changed in the 1990s, when Thompson identified a bonobo population in the southern extremity of their distribution range, inhabiting a transitional ecotone between moist forests and savannahs [45], [46]. Her discovery changed our perception of the ecological limit of the species range, but bonobos within this habitat remained poorly studied. In 2005, a new population living in the forest-savannah mosaic of western Democratic Republic of Congo (DRC), this time in the western extremity of the distribution range, was documented by the local NGO Mbou-Mon-Tour and by an extensive survey conducted by the World Wide Fund for Nature (WWF) [47], [48]. A study of bonobo genetic diversity across their entire distribution range has indicated that this population has probably been isolated from other populations since the Pleistocene [49]. This finding, combined with the fact that forest-savannah mosaics show large ecological variability compared to dense forests, suggests that this population could exhibit unique behavioural and ecological features. The region is characterized by high spatio-temporal variation in food availability. The home ranges of the local bonobos include forest patches of various shapes and sizes interspersed with numerous micro-habitats. In addition to this geographically patchy distribution of resources, periods of high scarcity in fleshy fruits were also documented. Studies in this region will provide us with an opportunity to better understand the full spectrum of bonobo adaptations. They also promise to clarify whether the grouping patterns of chimpanzees and bonobos reflect evolutionary adaptations or are reflections of current specific short-term environmental contexts.

Such research is also essential in the current context of the rapid human-engineered modification of the global landscape. The forests of the Congo Basin are being cleared or degraded at a rapidly increasing rate [50], and climate change could modify the pattern of rain seasonality in the region. Both factors are likely to induce larger spatio-temporal variation in the availability of food for great apes and other wildlife species. While some studies have already pointed out the effects of habitat fragmentation and related human activities on declines in ape density [29], [51], we still have a poor understanding of how variation in food availability might impact the population densities and social organization of great apes. In order to address the questions, we must improve our knowledge on both the population dynamics and on social structures for each species across their distribution range. Given that unhabituated great apes are elusive and that direct observations of them in their forest habitats are generally impossible, this can be achieved only by developing a systematic methodology which can be applied to study unhabituated populations.

In this study we present the first precise estimate of bonobo densities for the Malebo region and investigate the population dynamics there over a period of years. We also provide the first analysis of bonobo grouping patterns in a forest-savannah mosaic by using a systematic methodology based on indirect observations using night nests. More precisely, we focus on the influence of environmental factors on nest group size, testing whether the high seasonality of fruit availability influences bonobo cohesiveness at night by using a predictor reflecting the availability of fleshy fruits at the time of the nest-building. We also include three predictors which are known to influence choice of nesting sites in dense forests in order to test their influence on nest grouping patterns in this new environment: the availability of fleshy fruits at nesting sites, density of preferred nesting trees and rainfall. Finally, we controlled for the influence of human activity. Our finding offers first insights into the socio-ecological traits characterizing bonobos living in a forest-savannah mosaic.

## Materials and Methods

### Ethics statement

This non-invasive research was conducted using only indirect signs of bonobo presence (nests) under the WWF-DRC research permit (RM441976, granted by the Minister of Foreign Affairs and International Cooperation of Democratic Republic of Congo). For the questionnaire survey, we used the Poverty and Environment Network (PEN) prototype questionnaire developed by CIFOR. The questionnaire was approved by the ethic committee of the Biology Department of the Unikin (University of Kinshasa) and was authorized to be performed through the WWF permit. We explained to each person to not answer to a question if they desired to do so. Before conducting each interview, the goal of the study was explained to the interviewees and we asked their verbal approval to the participation of the study before starting (written consent was not asked for as most of the people are illiterate).

### Study site

**Figure 1:**
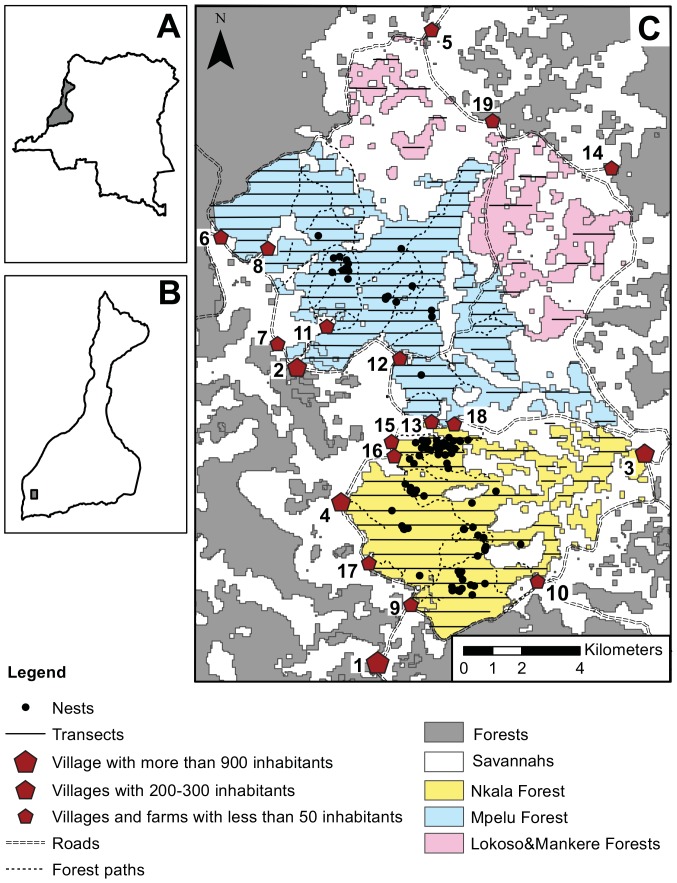
Map of the study site (16.41–16.56°E, 2.45–2.66°S, West DRC). A. Location of the Lake Tumba landscape in Democratic Republic of Congo. B. Location of the study site inside Lake Tumba landscape. C. Map of the study site. Forests are indicated in grey and savannahs in white (the map is based on a non-supervised classification – RED and IR on a Landsat7 (2007)). To represent the further subdivisions we made of the area, we coloured in yellow and blue the two suspected home ranges of bonobo communities habituated by WWF-DRC. Pink indicates the forest patches of re-colonizing *Uapaca sp.* Villages are depicted as red pentagons. Number 19 represents the WWF-base. Parallel dashed lines indicate the roads surrounding the study site, whereas dotted lines indicate the main forest paths. Vertical solid lines depict the 114 line transects (179.1 km) travelled in 2011, 2012 and 2013, and the nesting sites visited for our nesting site study are depicted as filled-in black points.

The study site is located in the South of the Lake Tumba landscape in western Democratic Republic of Congo, close to the WWF Malebo research station, in forests contiguous to Nkala and Mpelu villages (16.41–16.56°E, 2.45–2.66°S, Figure 1). This region can be characterized as a forest-savannah mosaic. The altitude ranges from 300 to 700 m [48], and the mean daily temperature fluctuates around 25°C [52]. Annual rainfall oscillates around 1500–1600 mm, and is interrupted by two dry seasons in February and July-August [48]. Forests mostly represent *terra firma* soil conditions and encompass various habitat types, i.e., re-colonizing *Uapaca sp.*, old secondary, mixed mature, old growth mono-dominant, riverine gallery and *Marantaceae* forests [48]. At the time of our data collection, the study site encompassed 170 km^2^, made up of 102 km^2^ of forest patches of various shapes and sizes which are connected by many corridors. Surrounding savannahs were mainly herbaceous and partially used for cattle ranching. Human activities and settlements were concentrated in the west side of the study area. Six villages and twelve farms were directly adjacent to the forest and plantations were located inside the forest. A bonobo population, probably made up of two communities, inhabited Nkala and Mpelu Forests, and has since 2007 been the subject of habituation and conservation programs by the WWF-DRC [48].

### Data collection

We collected data between April 2011 and August 2013 with the help of local assistants and with the support of WWF-DRC. In order to estimate bonobo density, we conducted three forest surveys in which we counted nests along line transects. In addition, we carried out a study of nest decay rates, which was necessary in order to convert nest densities into densities of bonobos. We monitored fruiting trees in order to acquire data on the seasonality of fruit availability, and we collected data on nesting sites to provide information on nest grouping patterns. For our subsequent analysis focussing on bonobo cohesiveness at night, we combined (i) nesting site data (nest counts, fruit availability at nesting sites and density of suitable nesting trees out of total trees available at the study site), with information on (ii) fruit availability in the forest, (iii) monthly rainfall at the study site and (iv) human activities in the forest, the latter achieved by administering a questionnaire to local villagers (Table 1).

**Table 1:** Summary of data collection.

Type of data	Period of data collection	Sample size of the dataset
Rainfall	May 2011 to June 2013	791 days
Tree abundance data	April to July 2011	8730 trees in 27ha of plots (15 plots of 1 ha, 48 plots of 0.25 ha)
Survey data	April to July 2011, Mid-March to Mid-July 2012, July to August 2013	114 line transects (total effort: 179.1 km)
Socio-economic data	Mid-May to mid-July 2012	201 people interviewed (see details in Table 3)
Nesting site data	May 2011 to May 2013	1872 nest trees at 104 nesting sites and 2259 control trees at 97 nesting sites
Nest decay time	May 2011 to May 2013	42 nesting sites (610 nests, part of the nesting site data)
Fruiting tree data	May 2011 to June 2013	672 trees between May 2011 and May2012, 4533 trees between May 2012 and May 2013

#### Rainfall

Between May 2011 and June 2013, rainfall was collected every twenty-four hours with a rain gauge at the Mbou-Mon-Tour farm (Figure 1, village number 16).

#### Tree abundance survey

In order to acquire baseline data on tree species abundance in the study site, we carried out a plot survey between April and August 2011. Sampling design was fully randomized and systematic using a 1 km^2^ grid. We made use of two plot sizes depending on their location in the forest: 0.25 ha for plots located less than 200 m from the forest edge (n = 48) and 1 ha for plots in the interior of the forests (n = 15). For each tree with a stem diameter at breast height (DBH, i.e., at 130 cm height) greater than 10 cm, we recorded the tree species and DBH (9730 trees in 27 ha in total). Four hundred and seventy-four samples of 178 tree species belonging to 44 families were registered in the herbarium and botanical library of the Université Libre de Bruxelles (“BRLU”), with reference IDs Bastin-Serckx#1-474.

#### Survey data

We delimited the size and shape of our study site based on WWF staff knowledge of bonobo home ranges in the Mpelu and Nkala forests and added connecting corridors. In April 2011, we conducted a pilot study during which we recorded all bonobo nests on reconnaissance walks (recces) to define the total sampling effort needed to perform a precise density estimation [53], [54]. Based on the results of the pilot study, we created a survey design with 114 transects running from west to east, spaced 500 m and of variable lengths, adding to a total of 179.1 km surveyed through the forest (Figure 1). We sampled transects in May to July 2011, mid-March to mid-July 2012 and June to August 2013. Due to external constraints, we were not able to visit some transects each year (see Table 2 for the exact annual total efforts). We systematically collected information on bonobo nests and recorded their perpendicular distances from the transects using a tape measure, following the methodology recommended in the IUCN guidelines [54] and Buckland et al. [53]. The three observers were trained together and used a consistent methodology.

**Table 2:** Area and total effort per year used for to estimate bonobo population density.

	Area (km^2^)	Total effort 2011 (km)	Total effort 2012 (km)	Total effort 2013 (km)
Global	93.84	130.1	179.1	175.5
Nkala	32.45	49.9	61.9	61.9
Mpelu	54.26	72.7	109.7	106.1
Lokoso&Mankere	7.13	7.5	7.5	7.5

#### Socio-economic data

Between May and June 2012, we collected socio-economic data in the six villages and the twelve farms surrounding the study site (Figure 1). We developed a questionnaire based on the “Poverty and Environment Network (PEN) prototype questionnaire” [55]. We randomly chose a minimum of 30% of adults in all local villages and farms [56]–[58]. We interviewed a total of 201 adults (Table 3) on their hunting and fishing activities as well as their collects of non-timber products. In addition, we asked about the frequency and location of each activity in the forest and the villager indicated the location of their activities on a forest map using the local names for each location in the forest.

**Table 3:** Socio-economic data.

		Population					Interviewed people			Interviewed people per activity				
		Nb household	Nb men	Nb women	Nb children	Total	Total	Men	Women	Hunters (M)	Fishermen (W)	Fishermen (M)	NTPC (W)	NTPC (M)
1	Nkoo	168	169	202	540	911	50	35	15	16	13	20	7	9
2	Mpelu	43	50	58	153	261	50	30	20	19	20	23	20	19
3	Lebomo	37	37	34	141	212	26	14	12	7	9	8	3	2
4	Nkala	34	36	49	110	195	39	21	18	7	18	14	16	10
5	Malebo	10	9	11	38	58	6	3	3	1	3	2	3	2
6	Mavula	10	10	12	25	47	6	3	3	3	3	2	3	1
7	Bosatore	7	5	7	22	34	2	1	1	1	1	1	1	1
8	Mokoabuo	6	5	8	17	30	4	2	2	1	2	1	2	1
9	Dispensaire de Nkoo	4	4	4	19	27	2	1	1	0	0	0	0	0
10	Lensiana	4	4	3	18	25	0	0	0	0	0	0	0	0
11	Biomengele	3	3	3	13	19	3	2	1	2	1	1	1	1
12	Ngandjele	3	3	6	7	16	2	1	1	0	1	0	1	0
13	Motsuemontore	2	2	4	9	15	2	1	1	1	1	0	0	0
14	Ezano	3	2	2	8	12	1	1	0	1	0	1	0	1
15	Mayi Monene	2	2	3	5	10	2	1	1	0	1	1	0	0
16	Mbou-Mon-Tour	4	4	4	2	10	2	1	1	0	1	0	0	0
17	Moza	1	1	1	6	8	2	1	1	0	1	0	1	0
18	Bosieli	1	1	1	5	7	2	1	1	1	1	1	1	0
	**TOTAL**	**342**	**347**	**412**	**1138**	**1897**	**201**	**119**	**82**	**60**	**76**	**75**	**59**	**47**

The numbers beside the village names were used to locate them on the study site in Figure 1. In the ‘Population’ part of the table, we present results of the village population census realized in 2012. The ‘Interviewed people’ part of the table indicates first the sampling effort for the socio-economic data collection (total per village and per gender). Finally, the ‘Interviewed people per activity’ part of the table gives the number of interviewed individuals (per village and per gender) who indicated that they regularly enter the forest for hunting, fishing or collecting non-timber products (‘NTPC’ =  non-timber products collect) and thus answered those parts of the questionnaire.

#### Nesting site data

Between May 2011 and May 2013, we gathered data on bonobo nesting sites (n = 104). For each month, we randomly selected one nesting site out of all of the sites located by the WWF trackers who were conducting daily follows of the bonobos for the WWF habituation program. We selected only nesting sites at which the trackers had been present at the evening nest-construction time to insure that we used only night nests, and we always collected nesting site data within 48 hours of nest building. During the May-June 2011 and May-June 2012 periods, we intensified data collection by gathering information on all of the nesting sites found by the WWF trackers. At each nesting site, we first explored the surrounding area to ensure that we had found all of the nests. We considered nests as being part of the same nesting site when the maximal distance between two nests did not exceed 30 m [6], [16]. We counted only fresh nests, i.e., nests built the previous night, with green leaves and traces of feces or urine [59]. For each tree containing a nest, from here on called a nesting tree, we recorded the species of tree (n = 1872). In order to further investigate nesting site characteristics, we randomly chose, in a subset of 97 nesting sites, a maximum of 30 control trees, which we identified to species level. These trees were distributed between the nesting trees, for a total of 2259 control trees.

#### Nest decay time

We conducted a nest decay rate study between August 2011 and May 2013, following previously validated methodology [54], [60]–[63]. We made repeated revisits to all nests identified as fresh during our nesting site study and assessed their conditions. For months where we characterized numerous nesting sites, we used only three randomly selected sites for the nest decay study. We made weekly visits to a total of 42 nesting sites containing 610 nests until the nests had disappeared [63]. At each visit we noted the degree of nest degradation according to the following categories: (i) new: only green leaves; (ii) recent: a mixture of green and brown leaves; (iii) old: only brown leaves; (iv) very old: brown leaves and the nest is losing its structure [59]; and finally, (v) disappeared: nest no longer recognizable [27]. We estimated mean nest decay time by using the method proposed by Laing et al. 2003 [61]. More specifically, we used the logistic regression model with left truncation. We bootstrapped the nest data (n = 1000) to estimate confidence intervals at 2.5%.

#### Fruiting tree data

Between May 2011 and May 2013, we recorded data on fruiting trees within 31 plots of 0.04 ha each, for a total of 1.24 ha (14 plots in the Nkala Forest and 17 plots in the Mpelu Forest). We randomly chose plot locations placed along the transects in order to facilitate our access to them. In November and December 2011, all trees with a DBH larger than 10 cm were marked, identified to the species level and their DBH was measured (n = 672). In May 2012, in order to improve our representation of fruiting trees, we added 14 additional plots (8.75 ha in total, from the tree abundance survey; Nkala Forest: five 1 ha plots and three 0.25 ha plots; Mpelu Forest: two 1 ha plots and four 0.25 ha plots). Every two weeks, we visited each of the plots and recorded which trees were fruiting by inspecting their crowns and counting fruits on the ground.

### Analytical methods

Prior to beginning our analysis of the social cohesion of bonobos at their nesting sites, we needed to estimate the density of bonobos in our study area, which was then modelled to understand their population dynamics over the years. Beside this, we modelled variation in fruit availability to investigate possible seasonal patterns. Finally, we modelled nest group size (i.e., the number of nests per site) according to fruit availability (across the entire home range and at the nesting site), ‘density of suitable nesting trees’, ‘rainfall’ and two control variables relating to human activities: ‘village influence’ and ‘human forest use’.

#### Bonobo population density estimate

We estimated the population density of bonobos in our study area from transect data. We walked 114 transects for 179.1 km of total effort, once per year in 2011, 2012 and 2013 (n = 1411 nests). Density was estimated using Distance 6.0 Release 2 [53], [64]. We divided the study site into three parts for the analysis to estimate the population density in the two presumed home ranges of the bonobo population living in the area, as documented from WWF data (the Nkala and Mpelu Forests), and the Uapaca sp. forest patches (Lokoso&Mankere) located at the north-east boundary of the study site (Figure 1). These young forest patches were surveyed during the three year period as we did not know if bonobos from the Mpelu community might have encompassed it within their home range. As we found no evidence of bonobo use of the area, in the end we did not consider it in the analysis to avoid underestimation of bonobo density. We post-stratified the dataset by year and by the three parts of the study site, then fitted a global detection function in order to obtain an estimation of numbers of individuals for each community. We derived a global estimation of the bonobo community size by weighting the data considering the size of the three parts of the study site. We truncated the data, keeping only nests for which the probability of detection from the transect was above 0.15. We tested different functions to model the data and chose the function that minimized the Akaike's Information Criterion (AIC, [65]). To convert bonobo nest density into density and number of bonobo individuals, we divided the nest density by the nest construction rate, the proportion of nest-builders and the nest decay time [53]. We used a nest construction rate of 1.37 per day [66] and considered the proportion of nest-builders in the population to be 0.75 [6]. The construction rate and proportion of nest-builders were taken from the literature, as these can only be estimated by following habituated individuals.

#### Variation in bonobo population density between years

In order to get a better understanding of variation in bonobo density between years, we analysed the transect dataset from each forest region surveyed in 2011, 2012 and 2013, and this independently for each presumed home range of the bonobo population (Nkala Forest: 31 transects, 61.9 km of total effort; Mpelu Forest: 72 transects, 111 km of total effort). The Lokoso&Mankere Forests were not taken into account for this analysis as we never observed nests in those forest patches during the surveys. We used a zero inflated generalized linear model with a negative binomial error structure and log link function [67], which enabled us to take into account the fact that the number of nests on transects was frequently zero but on some transects we occasionally found rather large numbers of nests. This type of model provides us with an option to independently model an excessive number of zeros together with count distribution, indicating which factors affected nest absence / presence on transects and which factors affected the number of nests encountered on transects. We used the specific year of the survey as a categorical predictor and we included its effect into the count and the zero inflation part of the model. We added an offset term to control for differences in transect length (for the zero inflated part this was 1/transect length; in both parts of the model we included the logarithm of the respective offset term). To account for spatial autocorrelation, we used the average of the residuals of all other transects derived from the full model and weighted by distance as an additional predictor. The weight function had the shape of a Gaussian distribution with a mean of zero (maximal weight at distance equals zero) and a standard deviation chosen such that the likelihood of the full model with the derived variable ('autocorrelation term’) included was maximized. The autocorrelation was only included into the count part of the model.

As an overall test of the effect of year, we compared the fit of the full model including year, the offset and the autocorrelation term with a null model comprising only the offset and the autocorrelation term. When the overall effect of year was significant, we tested which part of the model was significant by comparing the full model with two reduced models lacking year, either in the zero inflated part of the model or in the count part of the model. For these model comparisons we used likelihood ratio tests [68]. Finally, the effect of year was assessed by looking at estimates and p-values in the significant part of the full model. As year was a factor, we releveled it to obtain comparisons between the years 2012 and 2013. All analyses were conducted using R [69] and the additional package pscl [70]. We investigated model robustness by excluding data points one by one, rerunning the model and determining model coefficients and the significance of model comparisons. This did not reveal any obvious influential cases.

#### Variation in fruit availability between years

To test whether fruit availability exhibited seasonality and varied between forests, we used a generalized linear model. We used the ‘availability of fleshy fruit' index calculated per forest every two weeks as response (n = 106). Fruit species considered for this index were derived by selecting tree species (i) eaten by bonobos at different study sites [71], [72] (Serckx unpublished data) or (ii) producing fleshy fruits [73]–[75]. For each fleshy fruit-bearing species, we calculated the fruit index as the proportion of fruiting trees and we multiplied this value by the basal area (in square meters per hectare) of the species for the forest in which the plot was located (total plot samples equals 11.25 ha for the Nkala Forest and 14.25 ha for the Mpelu Forest, from data acquired in the tree abundance survey). Fruit indices of all fleshy fruit species were summed to obtain the fruit availability index used as response in the model. As our response did not follow a normal distribution, we used a function (powerTransform from the R package ‘car’[76]) to estimate a normalizing transformation of the residuals. This function reveals a parameter that makes the residuals from the regression of the transformed response (here the fruit availability) on the predictors as close to normally distributed as possible. We used as predictor the ‘date’ at which fruit availability was calculated. ‘Date’ was converted to a circular variable and its sine and cosine were included into the model to estimate seasonal patterns. We used ‘forest’ as a categorical predictor to check for differences in fruit availability between the two forests. To test whether the effect of season differed between the two forests we also included the interaction between these two predictors into the model. To account for temporal autocorrelation, we used the average of residuals of all other values of fruit availability derived from the full model and weighted (with the same function as for the previous model) by temporal distance as an additional predictor. After running the model, we checked various model diagnostics (Cook's distance, dfbetas, dffits, leverage and Variance Inflation Factors) and none of these indicated obvious influential cases or outliers or collinearity problems. Inspection of a qq-plot of the residuals and residuals plotted against fitted values indicated no obvious violations of the assumptions of normally distributed and homogeneous residuals.

As an overall test of the effect of seasonality we compared the fit of the full model including sine and cosine of the date, forest, their interaction and the autocorrelation term with a null model comprising only the forest and the autocorrelation term. To determine whether the effect of seasonality was the same in both forests, we compared the full model with a reduced model lacking the interaction. As the interaction was not significant, we removed it from the model and then tested the effect of seasonality by comparing this new model with a null model lacking date. Both comparisons were performed with an F-test. Finally, the effect of forest was obtained from estimates and p-values in the model lacking the interaction with season. All analyses were conducted using R [69] and the additional package car [76].

#### Effect of fruit availability on bonobo social cohesiveness

To test which factors affected nest group size, we ran two models, one assuming we had one bonobo community (Model 1), and the other assuming two bonobo communities (Model 2). The same predictors were used in both models, and community size (log transformed) was incorporated as an offset term. We used generalized linear models with negative binomial error structure and log link function. We excluded data from the beginning of May 2011 as some predictors were not yet available for this period. The dataset included 90 nesting sites (1439 nests) and we used nest count per nesting site as response.

We included three predictors to estimate the effects of environmental variables. We first incorporated the ‘density of suitable nesting trees’. This predictor gives the density of tree species preferred by bonobos for nest-building. To calculate this, we compared the distributions of individual nesting trees (n = 1872) with their abundance in the forest (n = 9730). Species for which identification to species level had not been achieved during the tree abundance survey were combined at the genus level in nesting tree abundance (5 species) and species not represented in the tree abundance survey were removed from nesting tree abundance (13 species). We first used a chi-squared test to check whether bonobos significantly preferred some tree species to build their nests (with the p-value determined based on permutation and not the chi square distribution, p<0.001). Binomial tests conducted separately for each species highlighted the preferred species (we use as significance threshold of p<0.05, n = 24 tree species). Finally, we calculated the density of those preferred species at each nesting site. The second predictor we used represents the ‘availability of fleshy fruits in the forest’ at the time when the nesting site was built. We selected the same fruit species we used in our model on fruit availability variation. According to the model, the predictor was determined for the entire study area (Model1) or separately for the two forests in which each community was presumed to live (Model2). We estimated a daily mean proportion of fruiting trees from the fruiting tree study by assigning for each date the value of the closest recorded proportion of fruiting trees. The fruit index was calculated as the mean proportion of trees bearing fruit during the 14 days before the nests were built multiplied by their basal area in either the study area (Model1, n = 9730) or in the forest (Model2, n = 4548 in the Nkala Forest and n = 5182 in the Mpelu Forest). Fruit indices of all fleshy fruit species were added to derive the fruit availability index. We then estimated ‘availability of fleshy fruits at the nesting site’. In this case, we used the same fruit species selected before, but we only took into account the fruit availability in the area around the nesting site, and, for each nesting site, we calculated the fruit index as the proportion of fruiting trees multiplied by their basal area at the nesting site and summed this for all fleshy fruit species.

We used the measure of ‘rainfall’ for the 30 days before nest building to control for seasonal variation in climate. To control for the possible influence of human activity on bonobo nesting sites, we first used the predictor ‘village influence’. To estimate this predictor, we summed for each nesting site the population size of each village divided by its distance to the nesting site. Secondly, we derived ‘human forest use’ from our questionnaire data by calculating the daily number of adults who could potentially enter the region of the forest where each nesting site was located in order to hunt, fish or collect non-timber products. Those activities were analysed by gender of the performer (e.g., hunting is only engaged in by men and ‘fish-scooping’ only by women). For each activity and for each village, we calculated the proportion of interviewed adults going in a forest region (‘prop_quest_adult’ in the formula). In order to obtain this index, we first estimated the probability of an adult entering a particular forest region (i.e., the daily frequency of the activity divided by the number of forest regions each person enters to engage in the activity) and then divided it by the number of interviewed adults performing the activity. We estimated the proportion of adults going to a forest region for each activity and each village and finally derived the overall index of human forest use for all villages and all activities: 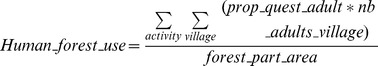
where nb_adults_village is the number of adults in a village (women or men according to the activity) and forest_part_area was the area of the forest region in square kilometers (used to account for differences in the sizes of the forest regions and to obtain values comparable between forest regions).

We further included an offset term to control for bonobo community size. Here, in contrast to the population density estimate, we used the number of nest-building individuals (log-transformed), which was also estimated using Distance 6.0 for each survey year. We used a nest-building individuals' estimate as we know that young bonobos do not make nest, instead sleeping in their mothers' nests. Here, we did not divide nest-density by the proportion of nest-builders (0.75 [6]) to obtain the number of nest-building individuals per forest region. For nesting site data collected between the periods of surveys, we did not have a bonobo community size estimate. To overcome this problem, we used community size estimated during the surveys before and after the nesting site was built and calculated a mean weighted by the time separating each survey and the build of the nest. We added an autocorrelation term, simultaneously taking into account temporal and spatial autocorrelation. For this, we used the average of residuals at all other nesting sites derived from the full model, weighted (with the same function as for the previous models) by spatial and temporal distances. This time we used two standard deviations, one for spatial and one for temporal autocorrelation, which were determined simultaneously.

All analyses were conducted using R [69] and the additional packages gtools [77], car [76], and MASS [78]. Prior to running each model, we checked that correlations between predictors were not an issue with a Spearman test and that all predictors had a symmetrical distribution. ‘Human forest use’ was log-transformed. All quantitative predictors were z-transformed to a mean of zero and a standard deviation of one to achieve more easily interpretable coefficients [79]. We inspected two model diagnostics: Variance Inflation Factors (which was not an issue) and leverage. As our data showed some potentially influential cases, we used a subset of our data for the analysis (n = 86 for both models). As the autocorrelation term was not significant, it was removed from the model for final results. After running the models, we corrected the AIC for small sample size. In order to test for the overall effect of the environmental variables (‘availability of fleshy fruits in the forest’, ‘availability of fleshy fruits at the nesting site’, ‘density of suitable nesting trees’ and ‘rainfall’), we compared the fit of the full model including all predictors, the autocorrelation term and the offset term with a null model comprising only the intercept, the two variables controlling for human activity, the autocorrelation term and the offset term (chi-square test).

## Results

### Bonobo density estimation

**Figure 2:**
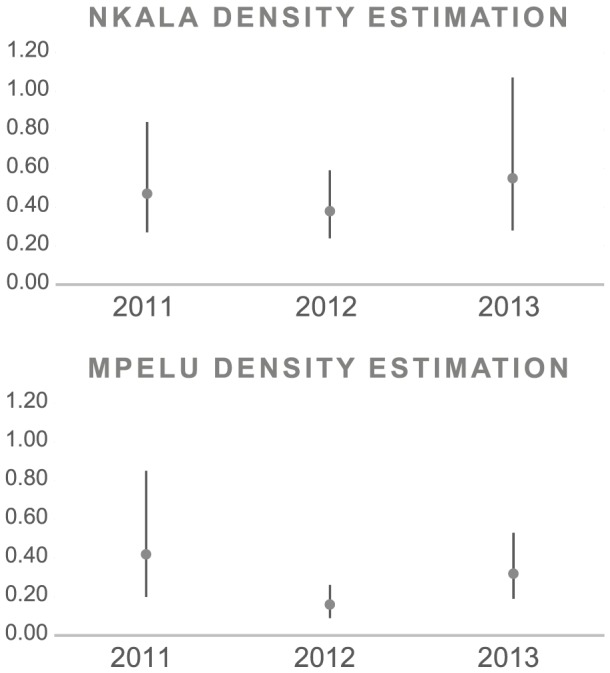
Bonobo population density over the three year period (2011, 2012 and 2013). Points represent the population density estimation, with lines added showing the lower and upper boundary of the 95% confidence interval.

**Figure 3:**
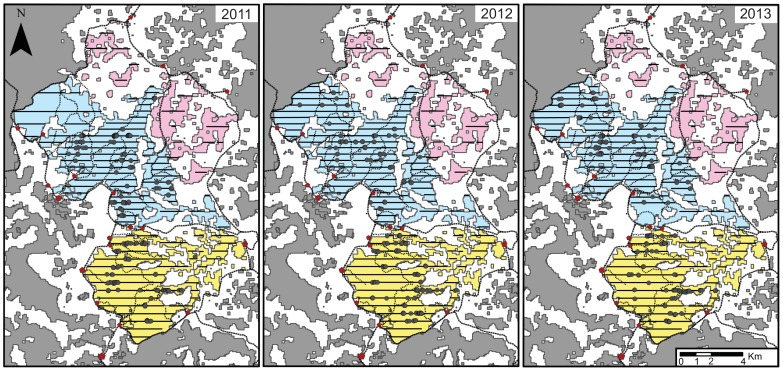
Map of the nests found on the transects during each survey (2011, 2012 and 2013). We here indicate nests as grey points. The different forest colours represent the area subdivisions used for our population density estimation. The transect lines have been added as well (see Table 2 for the exact total effort of each year). Villages, roads and main forest paths are represented as in Figure 1.

Logistic regression revealed a mean nest decay time of 183 days (range: 179-188 days). In order to estimate bonobo density, we truncated our transect data at 35 m perpendicular distance, which led to a decrease in the number of nests from 1411 to 1341. We modelled the data with a half normal cosine function. The effective strip width (‘ESW’) was 19.1 m with a mean probability of detection of 0.55 (Table 4). For 2011, 2012 and 2013, respectively, we estimated bonobo density to be 0.63, 0.51 and 0.55 individuals per square kilometer in the Nkala Forest and 0.56, 0.21 and 0.32 individuals per square kilometer in the Mpelu Forest (Figure 2). As results showed large differences between years, especially for Mpelu community, we carried out further analyses to better understand the reason for these variations (Figure 3).

**Table 4:** Bonobo population density and number of adult individuals in 2011, 2012, 2013, respectively, as estimated with Distance 6.0.

	% CV	D	D LCL	D UCL	N	N LCL	N UCL
Global estimation^1^	14.38	0.41	0.32	0.56	39	30	53
Mpelu 2011	36.5	0.56	0.27	1.13	31	15	61
Mpelu 2012	27.08	0.21	0.12	0.35	11	7	19
Mpelu 2013	26.24	0.32	0.19	0.53	17	11	28
Nkala 2011	27.85	0.63	0.36	1.12	20	12	36
Nkala 2012	22.65	0.51	0.32	0.79	16	11	25
Nkala 2013	33.54	0.55	0.28	1.07	17	9	35
Lokoso&Mankere 2011	0	0	0	0	0	0	0
Lokoso&Mankere 2012	0	0	0	0	0	0	0
Lokoso&Mankere 2013	0	0	0	0	0	0	0

We modelled data with a half normal cosine function and used a truncation distance of 35 m. We obtained an effective strip width (‘ESW’) of 19.1 m and a mean probability of detection of 0.55. %CV =  Coefficient of Variation for the density estimate. D = density estimate. D LCL =  Lower confidence limit (95%) of the density estimate. D UCL =  Upper confidence limit (95%) of the density estimate. N =  community size estimate (number of adults). N LCL =  Lower confidence limit (95%) of the community size estimate. N UCL =  Upper confidence limit (95%) of the community size estimate (^1^the global estimate was derived by weighting data with the area of the three parts of the study site).

In the Mpelu Forest, we found an overall effect of year on nest density (model including year vs. null model, likelihood ratio test, chi square = 9.59, df = 4, p<0.05). More precisely, our results did not show an influence of year on the distribution of nests on the transects (model with year vs. reduced model lacking year only in the zero inflated part of the model, likelihood ratio test, chi square = 3.71, df = 2, p = 0.16), but highlighted as a trend the influence of year on the number of nests on transects (model with year vs. reduced model lacking year in the count part of the model, likelihood ratio test, chi square = 5.03, df = 2, p = 0.08). We further conducted pairwise comparisons between years, looking at the nest count portion of the model. Results indicated a trend showing a decrease in nest density between 2011 and 2012 (Table 5, p = 0.050), a significant increase between 2012 and 2013 (Table 5, p = 0.043) and no significant difference between 2011 and 2013 (Table 5, p = 0.913). On the other hand, we did not find any effect of year on nest density in the Nkala Forest (model including year vs. null model, likelihood ratio test, chi square = 3.27, df = 4, p = 0.51).

**Table 5:** Variation in the density estimate between years (results of the zero inflated Generalized Linear Model with a negative binomial error structure and log link function).

Count model (with 2011 in the intercept)				
	Estimate	Std. Error	z value	P value
**Intercept**	1.172	0.311	3.763	**<0.001**
**Year 2012**	−0.685	0.349	−1.958	**0.050**
**Year 2013**	0.038	0.354	0.109	0.913
**Ac.term**	0.467	0.146	3.181	**0.001**

Count model (with 2012 in the intercept)				
	Estimate	Std. Error	z value	P value
**Intercept**	0.487	0.337	1.445	0.148
**Year 2011**	0.685	0.349	1.958	**0.050**
**Year 2013**	0.723	0.358	2.022	**0.043**
**Ac.term**	0.467	0.146	3.181	**0.001**

‘Year’ was dummy coded. The intercept represents 2011 in the first table and 2012 in the second table.

### Variation in fruit availability between years

**Figure 4:**
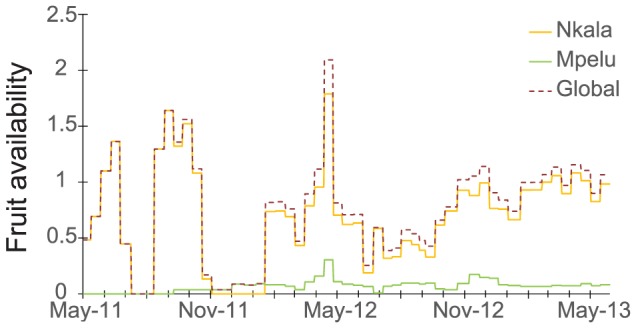
Global fleshy fruit availability and distribution per year. This figure represents the daily fleshy fruit availability of the forest used for the cohesiveness model in the Nkala and Mpelu Forests (used in Model2), as well as the sum for both forests together (‘Global’, used in Model1).

**Figure 5:**
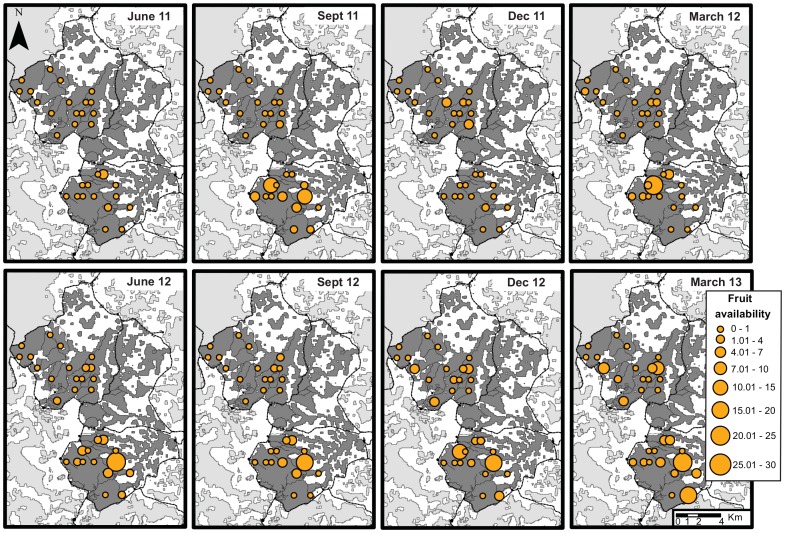
Maps of fleshy fruit availability and changes over time for each fruit tree plot. The availability of fleshy fruit was calculated as the sum of the basal areas of the fruit-bearing observed in the plot, which was then divided by the plot area to reveal an index per hectare, similar to the fleshy fruit availability calculated for the nesting sites and the forest. Here we show a representation of the three-month mean. Circle sizes are proportional to the availability of fleshy fruits in the plots. Villages, roads and main forest paths are represented as in Figure 1.

**Figure 6:**
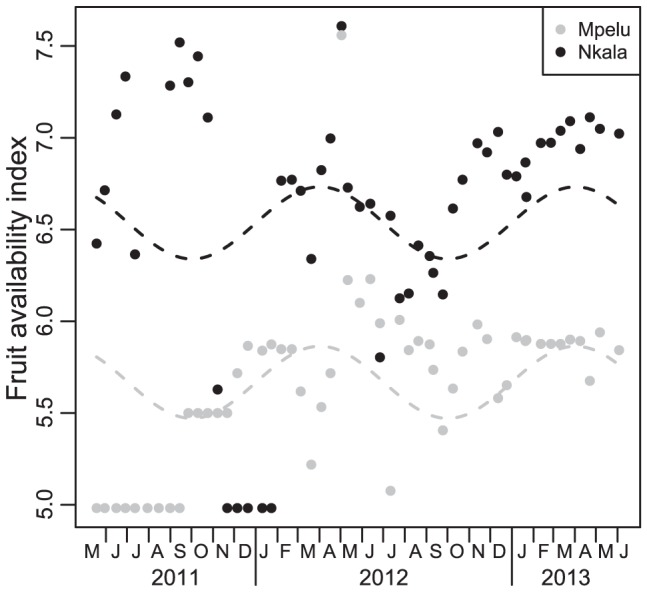
Temporal variation of fleshy fruit availability in ‘Nkala’ and ‘Mpelu’ forest. The results from the Nkala Forest are indicated in black and Mpelu in grey. Points represent fleshy fruit availability index every two weeks. Dashed lines indicate the fitted model. The dotted lines have the same amplitude as the model and revealed no significant interaction between seasonality and forest (F_2,106_ = 1.90, p = 0.15). The effect of seasonality was significant (F_2,106_ = 3.51, p<0.05), and fruit availability clearly differed between the two forests (estimate = 0.868, SE = 0.105, t-value:8.268, p<0.001).

Fruit availability showed high variation between the two years of data collection (Figure 4), with large differences between plots as well (Figure 5). Analysis revealed that the overall effect of seasonality was significant (model including date, forests and their interaction vs. model including only forest, F_2,106_ = 3.14, p<0.05). The pattern of seasonality was similar in both forests (model including the interaction vs. model without it, F_2,106_ = 1.90, p = 0.15) and was significant in both forests (model with date and forest vs. model lacking date, F_2,106_ = 3.51, p<0.05). We also found that fruit availability was significantly higher in the Nkala Forest (Table 6, p<0.001). A representation of fruit availability with the fitted model is presented in Figure 6.

**Table 6:** Variation in fruit availability between years (result of the Generalized Linear Model with a Gaussian error structure).

	Estimate	Std. Error	t value	P value
**Intercept**	5.668	0.074	76.285	**<0.001**
**Nkala Forest**	0.868	0.105	8.268	**<0.001**
**sin (date)**	0.197	0.074	2.649	**0.009**
**cos (date)**	−0.003	0.074	−0.039	0.969
**Ac.term**	0.251	0.053	4.753	**<0.001**

Here we show the results of the model, with sine and cosine of date representing seasonal patterns, and forest and an autocorrelation term (Ac.term) as predictors. Results indicate that forest had a significant effect on fruit availability (Mpelu Forest is included in the intercept as it is a categorical predictor). A significant effect of the seasonal pattern was obtained by comparing this model with a null model lacking date (F_2,106_ = 3.51, p<0.05).

### Effect of fruit availability on bonobo social cohesiveness

**Figure 7:**
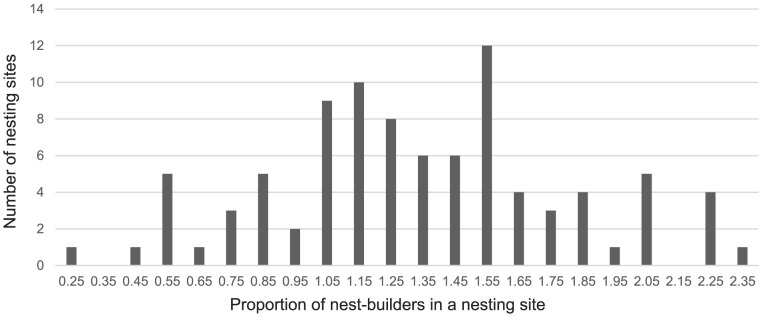
Frequency of the proportion of nest-building bonobos present at each nesting site. We calculated the proportion of nest-building bonobos as the number of nests divided by the estimated number of nest-builders in the community.

**Figure 8:**
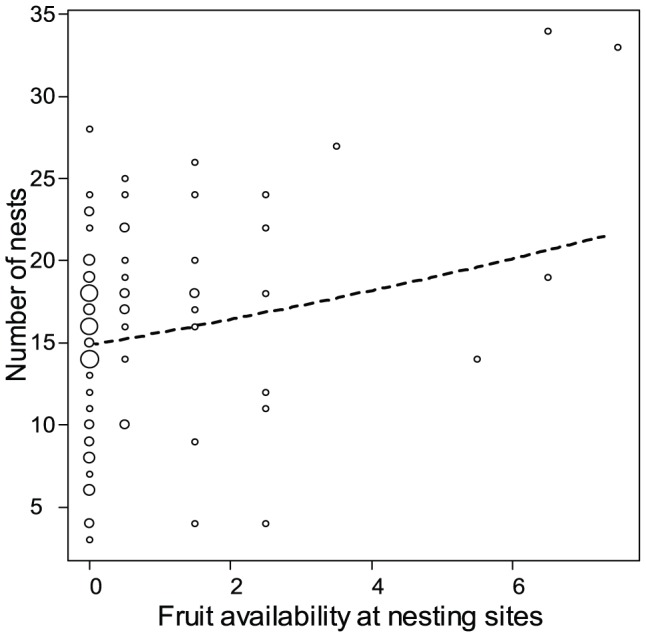
Number of nests at a nesting site as a function of fruit availability. The area of the circles indicate the number of nesting sites per fruit availability and number of nests. The dashed line represents the fitted model.

Because bonobo density varied between years in the Mpelu Forest, we hypothesized that, rather than having two communities within the study site, we might actually have one single large community, which regularly subdivides into smaller subgroups. Moreover, nest counts in some nesting sites were larger than the independently-derived estimation of the numbers of nest-building individuals in the two purported separate communities, suggesting that the two subgroups (if indeed they are separate subgroups) might sometimes aggregate (Figure 7, 80% of nesting site observations present a ratio of the nest count divided by the estimation of nest-building individuals equals or above 1). For this reason, when we analysed the effects of environmental factors on bonobo cohesiveness at nesting sites, we first compared two models representing either a single community hypothesis or a two community one. We compared the AICs of the two models to derive the most likely community composition of the area. Results clearly indicated that the ‘two community’ hypothesis better explains the number of nests in the nesting sites (comparisons of the AIC of the two models, Model1: one community, AIC = 572 vs. Model2: two communities: AIC = 539). The overall effect of the environmental variables was significant in the two communities model (χ^2^ = 11.42, df =  4, p<0.05), and the model revealed that ‘fruit availability at the nesting site’ significantly influenced the number of nests in nesting sites (Table 7, p<0.05, Figure 8) along with a trend for a positive influence of ‘density of suitable nesting trees’ (Table 7, p = 0.050), but no influence of the ‘fruit availability in the forest’ (Table 7, p = 0.249). ‘Rainfall’ and the two predictors of human activity did not reveal any influence on the nest grouping patterns at the study site (Table 7).

**Table 7:** Effect of the environmental factors on nest community size (Generalized Linear Model Models with negative binomial error structure and log link function).

Two community hypothesis				
	Estimate	Std.Error	z value	P value
**Intercept**	0.287	0.035	8.304	**<0.001**
**Density suitable nesting trees**	0.070	0.036	1.960	**0.050**
**Fruit availability forest**	0.049	0.043	1.152	0.249
**Fruit availability nesting site**	0.109	0.046	2.381	**0.017**
**Village influence**	0.011	0.065	0.177	0.860
**Human forest use**	0.045	0.040	1.117	0.264
**Rainfall**	−0.016	0.041	−0.388	0.698

This table shows the result of the ‘two community’ hypothesis and the result of ‘One community’ hypothesis are not shown. P-values of the predictors revealed a significant influence of ‘fruit availability at the nesting site’ and a trend for a positive influence of ‘density of suitable nesting trees’ on the number of nests at a nesting site. The autocorrelation term was removed from the model as it was non-significant (p = 0.42).

## Discussion

The primary aim of this study was to investigate the effects of fruit availability on bonobo cohesiveness at nesting sites in the forest-savannah mosaic of western DRC. This is a particularly interesting environment in which to study this phenomenon given its large spatio-temporal variation of resource availability. As expected, results indicated that fruit availability followed a seasonal pattern but also differed significantly in the various sampled forests (Figure 6). This latter finding was not surprising given that forest patches are composed of numerous micro-habitats in which the dominance of certain tree species varies. It also suggests that bonobos should be obligated to adapt their foraging strategies (daily travelled distance, party size, etc.) to the specific characteristics of their home range forests. Global fruit availability, however, did not seem to influence night grouping patterns, as only the availability of fruits at nesting sites was related to bonobo community cohesiveness (Table 7). Finally, our study of bonobo population density provided the quite unexpected result that community size varied between years in one of the studied forests (Mpelu). Additional long term studies including direct observations of bonobos would help determine whether or not this pattern is unique to our study region or is a common one for bonobos across their range.

Several competing hypotheses can be proposed to explain this surprising temporal variation in bonobo density. First we could argue that the variation is merely the result of sampling artefacts (nests) instead of the bonobos themselves. This is unlikely, however, as the transect effort was similar for each year of the study (81.4 km, 111 km and 108.9 km for respectively 2011, 2012 and 2013), and the models of bonobo density variation gave accurate results. Those models indicated that the bonobos tended to use the same areas for nesting year after year (the effect of year was non-significant in the zero inflated part of the model, p = 0.15), even when their average community size varied. This clumped distribution of nests on some of the transects suggests that bonobos maximize their access to feeding ‘hot-spots’. This interpretation is supported by the results of another study which was carried out in the area, which found that variation along transects in bonobo nest density was explained by the availability of fleshy fruits and edible terrestrial herbaceous vegetation, as well as by previous evidence of nests (i.e., the nest density of a previous survey; Serckx *et al*. in prep). A second hypothesis that might explain the variation in bonobo density is the impact of poaching or disease events, two major threats to bonobo survival across their range [80]. Although this might explain the apparent population decrease between 2011 and 2012, but such events are nearly impossible to observe in the field (Hohmann pers. comm.) and were not observed by WWF trackers or the local community. Nevertheless the apparent high increase in bonobo density between 2012 and 2013 (from 0.21 to 0.32 ind/km^2^, corresponding to 6 individuals being added to the community; Table 4) and the non-significant difference in bonobo density between 2011 and 2013 (Table 5) suggest that the poaching / disease hypothesis is insufficient to explain the variation in community size at our site. Finally, the density variation might have a very simple explanation: perhaps the study site did not encompass the entire home range of both communities. Previous studies have shown seasonal and yearly variations in home range size [81], with overlaps between community home ranges of the same bonobo population [82], [83]. Also fruit availability in the Mpelu Forest was significantly lower than in the Nkala Forest (p<0.001, Table 6) during the entire study, suggesting that the Mpelu community might have to adapt their foraging strategies to relative food scarcity. This hypothesis is reinforced by our observation of bonobo signs in 2013, at the north-west boundary of the study site, suggesting they also use the western forest patches which we did not survey. The home ranges of the bonobos which were estimated at the beginning of the WWF habituation program may then need to be readjusted to take into account the new picture painted by cumulative years of density estimation and direct observations as habituation progresses.

Our results show that the overall food had no clear influence on night time grouping patterns, as we found only a significant influence of local fruit availability on nest numbers, but no influence of the overall fruit availability of the forest (Table 7). This finding is consistent with the results of previous studies in the dense forests of central DRC, in which bonobos were found to aggregate at night close to food ‘hot-spots’ (Fruth pers. comm.) and in which fruit availability did not explain party size [16], [30]. Our model indicated a trend for the density of nesting-tree species having a positive influence on bonobo grouping patterns. Bonobos are known to have preferences for certain tree species with the right leaf sizes and branch resistance in which to build their nests [6], [16] (Fruth pers.comm.). The high abundance of these nesting-tree species in the Nkala and Mpelu forests probably explains why this factor had only a weak influence on bonobo social cohesiveness. In addition, the absence of a significant impact of human activities on the bonobos nesting patterns should be interpreted with caution and may be restricted to our study site, where the local ethnic group does not hunt bonobos due to ancestral taboos [48].

Our results, however, include the unexpected discrepancy that the nest counts at nesting sites were often higher than the nest-building community size estimated in the home range of the respective bonobo community (Figure 7). Studies of bonobos and chimpanzees have generally shown opposite results, reflecting the fact that all community members, in both species, commonly do not sleep together at one nesting site [16]. This particular result may be due to an underestimation of the number of nest-building individuals at our study site. First, when we estimated bonobo density, we used a nest production rate obtained at another study site. Second, as we have already highlighted when explaining the yearly variation in the population density of the Mpelu community, we probably failed to account for the entire home range of the two communities. Since we calculated the number of individuals per community by multiplying the population density of each community by the respective home range area, our underestimation of the home range sizes likely led to a subsequent underestimation in the community size. This explanation is supported by the direct observations of bonobos by WWF trackers who made regular counts and produced slightly higher population estimates than our study (WWF estimates in 2013: 21 individuals in Nkala and 40 individuals in Mpelu although Mpelu community can be divided in two sub-groups – Lahann pers. comm. – vs. 17 individuals in each community in our study, Table 4). It is possible, however, that the bonobos may have on occasions built more than one nest prior to sleeping, or they may have reused nesting sites over successive nights. Previous studies carried out in dense forests have also shown that separate bonobo sub-communities sometimes join together into one larger community [31], [82]. This might explain large variation in nesting site size, but the results of modelling clearly favour the hypothesis that two separate communities are present in our study region. On the other hand, in our study we probably over-represented larger nest groups as we used only nesting sites previously located by the WWF trackers, who, when they had to make a choice, preferentially followed the largest bonobo parties for the purpose of habituation. Caution is therefore required when extrapolating average nest group size from our results, and we do not do it here. Overall, however, our findings still suggest that bonobos tend to aggregate as the evening approaches (Figure 7), as bonobos from dense forests do [16], [30] (Fruth pers. comm.), and despite the fact that they have to deal with high variation in fruit availability in the forest-savannah mosaic. This supports the hypothesis that chimpanzee and bonobo grouping patterns have been formed by a long process of ecological and behavioural adaptations rather than reflecting current environmental variation [37].

This study provides the first data on bonobo social cohesiveness in a forest-savannah mosaic, and also suggests interesting new approaches for conservation programs. First, the importance of food ‘hot-spots’ indicates that well-defined areas should be selected and made the focus of the integrated management of conservation programs in reserves or logging concessions. Secondly, our results indicating the importance of yearly variation in home range size underlines the importance of establishing connections between forests. This is important not only for the home range adaptations of bonobos to changing fruit availability, but for female migration between communities at maturity, both of which are crucial for the long term survival of the species.

Our overall conclusions will need to be confirmed by direct observations, but strongly indicate that bonobos remain highly socially cohesive in the forest-savannah mosaic of western DRC. That this is the case in a region where fruit availability shows high variability in over time and across space, suggests that the grouping patterns of the species are not driven by current environmental conditions. However, further studies using systematic methodology are required in order to compare the influence of fruit availability on bonobo and chimpanzee social cohesiveness across all their habitat ranges. This should allow us to determine whether the differences in grouping patterns between bonobos and chimpanzees are intrinsic to the species. Do they result from specific evolutionary events in the context of past environmental contexts or do they mainly reflect current variation in food availability in the ranges of chimpanzee and bonobos? Further research should also be conducted over larger spatial scales and in human-modified habitats, such as in logging concessions, in order to shed light on the plasticity of social structure in both species, in particular in regard to the potential impacts of human global landscape modification, e.g. resource-extraction, the opening of forests, forest fragmentation and / or increased human agricultural activity. In addition to those results, we have also presented here the first precise density estimation of bonobos for this unique habitat-type, which has until now been one of the least well-investigated ecotones within the bonobo range. Our estimation of the bonobo population density in this area falls within the range of population densities found across Congo Basin Cuvette [84], suggesting that the Lake Tumba Landscape harbours a significant population of bonobos and urgently requires further surveys in order to allow us to more accurately estimate the global bonobo population size [80]. Furthermore, our results suggest that bonobos living in forest-savannah mosaics may be obligated to adapt their foraging strategies to the availability of fruit by significantly altering their home ranges. This finding should be investigated further with regards to its consequences for the conservation of this species within fragmented habitats. Finally, we would like to suggest that, whenever possible, researchers make use of data covering a period of several years when modelling great ape densities, as this should enable to better interpret changes in communities densities which are of vital importance when making species or site comparisons.

### Public Access to Data

All raw data from the survey on apes are archived into the IUCN/SSC A.P.E.S. database (http://apes.eva.mpg.de/) [85].
